# Anti-Anxiety Effects of Essential Oil Microemulsion in Chronic Unpredictable Mild Stress-Induced Rats: Preparation, Characterization, and Mechanisms

**DOI:** 10.3390/molecules30122652

**Published:** 2025-06-19

**Authors:** Wenxia Tang, Pan Jiang, Ke Hu, Duo Mei, Qinghao Jiao, Yan Li, Yanping Deng, Jun Wang, Ran Gao, Xin Chen, Jie Yu

**Affiliations:** School of Life Science and Technology, Wuhan Polytechnic University, Wuhan 430023, China; tangwenxia2022@163.com (W.T.); 15906803974@163.com (P.J.); huke2077@163.com (K.H.); 18672323259@163.com (D.M.); 18899686667@163.com (Q.J.); 17764061495@163.com (Y.L.); dengyanping20@126.com (Y.D.); wangjun202502@126.com (J.W.); h2025126@126.com (R.G.)

**Keywords:** herbal, microemulsion, aromatic plant essential oil, anxiety, sedative and hypnotic effects, network pharmacology

## Abstract

Anxiety disorders, as common neurological diseases in clinical practice, often coexist with depression. Epidemiological surveys indicate that approximately 85% of patients with depression exhibit significant anxiety symptoms. This comorbid state not only exacerbates clinical symptoms but also leads to treatment resistance and prolonged disease duration. This study innovatively developed a compound aromatic plant essential oil (EO) formulation with remarkable anxiolytic and antidepressant effects and systematically elucidated its mechanism of action. The study found that the essential oil formulation, administered via inhalation, could significantly improve behavioral abnormalities in animals subjected to the chronic unpredictable mild stress (CUMS) model, specifically manifesting as (1) the reversal of stress-induced weight gain retardation; (2) a significant increase in sucrose preference; (3) an increase in the total distance of spontaneous activity; and (4) the prolongation of exploration time in the open arms of the elevated plus maze. Neuropathological examinations confirmed that the formulation could effectively protect the structural integrity of hippocampal neurons and alleviate CUMS-induced neural damage. In terms of mechanism of action, the study revealed that the formulation regulates the neurotransmitter system through multiple targets: (1) the upregulation of serotonin (5-HT) and γ-aminobutyric acid (GABA) levels; (2) the downregulation of glutamate (GLU) concentration; and (3) key targets identified via network pharmacological analysis, such as ESR1, STAT3, and PPARG. These findings provide molecular-level evidence for understanding the neuromodulatory effects of aromatic essential oils. Pharmaceutical formulation studies showed that the oil-in-water (O/W) type compound essential oil microemulsion, prepared using microemulsification technology, has a uniform particle size and excellent stability, maintaining stable physicochemical properties at room temperature for an extended period, thus laying a foundation for its clinical application. This study not only validates the practical value of traditional medicine but also provides new ideas for the development of novel anxiolytic and antidepressant drugs, achieving an organic integration of traditional experience and modern technology.

## 1. Introduction

Anxiety disorders, characterized by excessive and uncontrollable fear and tension, represent one of the most prevalent neurological conditions in clinical practice, including panic disorder, generalized anxiety disorder, and obsessive-compulsive disorder [1,2,3]. Epidemiological studies indicate a lifetime prevalence of 33.7% and a global prevalence of 3.6%, posing a significant public health burden worldwide due to high incidence rates, disability rates, and their association with suicidal behavior [4,5,6]. Notably, anxiety and depression frequently co-occur, with clinical diagnoses showing that patients with depression often present comorbid anxiety symptoms, which not only prolongs treatment duration but also increases therapeutic difficulty [7,8,9]. Currently, benzodiazepines (BDZs) and selective serotonin reuptake inhibitors (SSRIs) remain first-line treatments for anxiety disorders; however, long-term use may lead to dependence, withdrawal reactions, and reduced tolerance [6,10,11,12]. Therefore, there is an urgent need to develop alternative therapies that have fewer side effects and sustained efficacy.

Aromatherapy, as a non-pharmacological intervention, has gained increasing attention in recent years for its potential to alleviate anxiety and stress. Essential oils—volatile extracts from aromatic plants—were first documented in the *Shennong Ben Cao Jing* (2nd century AD), with their use traced back to as early as 5000 BCE (Before Common Era) in ancient Egypt [13,14,15]. Modern research confirms their sedative, analgesic, anxiolytic, and antidepressant properties [12,16]. However, single essential oils often require high doses to achieve significant effects, prompting the development of compound formulations to enhance synergistic interactions and therapeutic efficacy [17,18,19].

In Traditional Chinese Medicine (TCM) theory, anxiety disorders fall under the categories of “depression syndrome”, “palpitations”, and “insomnia”, with their pathogenesis closely linked to emotional dysregulation and visceral dysfunction [20,21,22,23]. A compound formulation comprising *Acorus calamus* L., *Santalum album* L., *Citrus limon* L. *peel*, and *Mentha piperita* L. has demonstrated effects in “soothing the liver to relieve stagnation, opening the orifices, and refreshing the mind” [11,12,24,25,26]. Specifically, *Acorus calamus* L. (Shi Chang Pu) is known for its mind-opening and intelligence-enhancing properties. Historical texts document its use for antidepressant, sedative, and hypnotic purposes [18,27,28]. *Santalum album* L. (sandalwood) regulates liver qi and relieves chest pain, with its essential oil exerting anxiolytic and neuroprotective effects by reducing oxidative stress and modulating inflammatory pathways [29,30]. *Citrus limon* L. *peel* (orange peel) is traditionally used to regulate qi, strengthen the spleen, and resolve dampness-phlegm and is often employed in TCM to disperse liver qi stagnation [31,32]. *Mentha piperita* L. (Peppermint) is particularly effective in soothing liver qi stagnation and clearing the head, significantly alleviating depressive and anxious symptoms [33]. Historical records indicate that these herbs were traditionally made into sachets to calm the mind and uplift the mood [27,34,35], suggesting their potential as a modern anxiolytic formulation.

**Figure 1:**
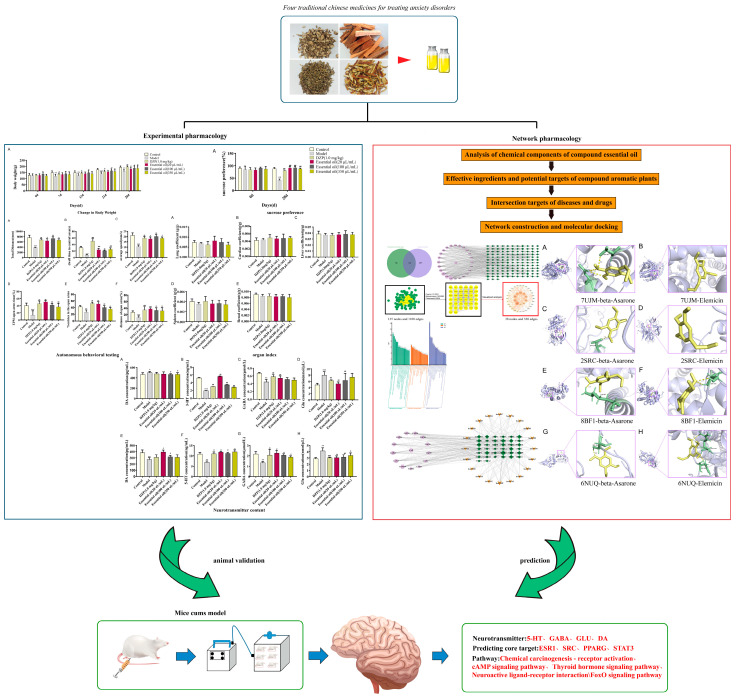
Schematic representation of the anti-anxiety effects of essential oil microemulsions in CUMS-induced rats. Data are represented as the mean ± SD (*n* = 6). * *p* < 0.05 ** *p* < 0.01, *** *p* < 0.001 vs. the control group, ^#^ *p* < 0.05, ^##^ *p* < 0.01, ^###^ *p* < 0.001 vs. the model group, ns indicates no significant difference.

Addressing the technical limitations of traditional essential oil preparations (e.g., high volatility and poor stability) [36], this study innovatively developed a compound aromatic essential oil microemulsion. Using the chronic unpredictable mild stress (CUMS) model, which effectively mimics human stress-related affective disorders [24,25,26], we systematically evaluated the anxiolytic effects of EOs through multidimensional approaches, including neurotransmitter analysis, network pharmacology, and molecular docking. This investigation elucidates the modulation of hippocampal GABA, 5-HT, and other neurotransmitter networks by EOs while characterizing component–target–pathway interactions at the molecular level. Our findings provide scientific evidence for modernizing TCM aromatherapy applications and establish a theoretical foundation for the development of novel anxiolytic agents. The overall study design is presented in Figure 1.

## 2. Results

### 2.1. Chemical Composition Analysis of Essential Oils

**Figure 2:**
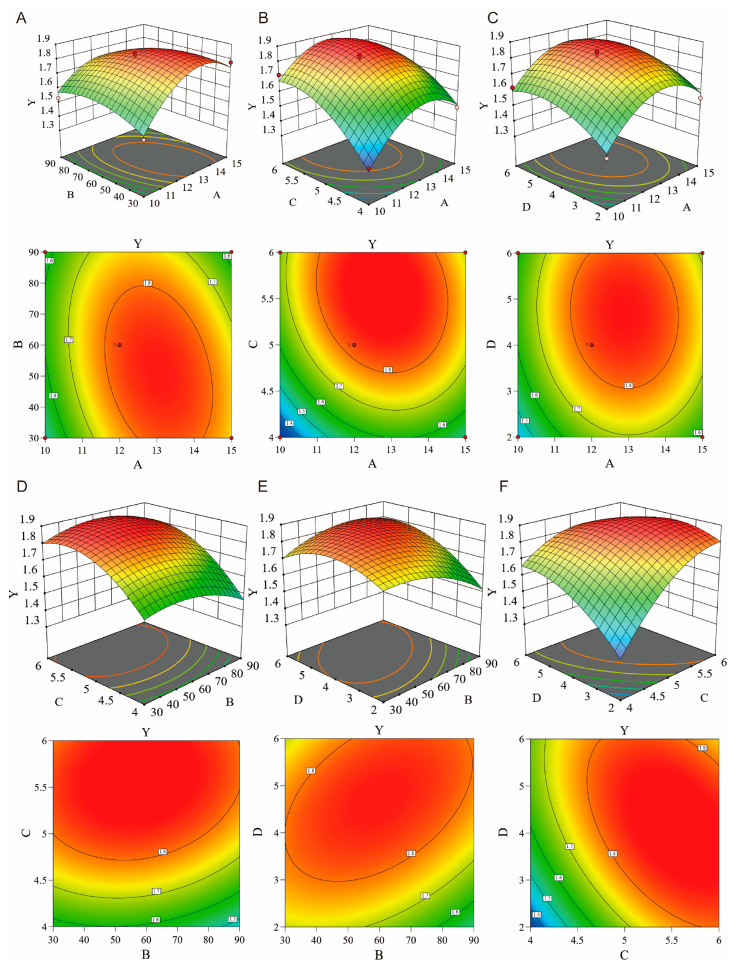
Three-dimensional and two-dimensional graphs of interaction diagram between factors. (**A**) Effect of solid–liquid ratio and ultrasonic time on extraction rate. (**B**) Effect of solid–liquid ratio and extraction time on extraction rate. (**C**) Effect of solid–liquid ratio and NaCl concentration on extraction rate. (**D**) Effect of ultrasonic time and extraction time on extraction rate. (**E**) Effect of ultrasonic time and NaCl concentration on extraction rate. (**F**) Effect of extraction time and NaCl concentration on extraction rate. (A: Solid liquid ratio (g/ml), B: Ultrasound time (min), C: Extraction time (h), D: NaCl concentration (%), Y: Extraction ratio (%)).

**Figure 3:**
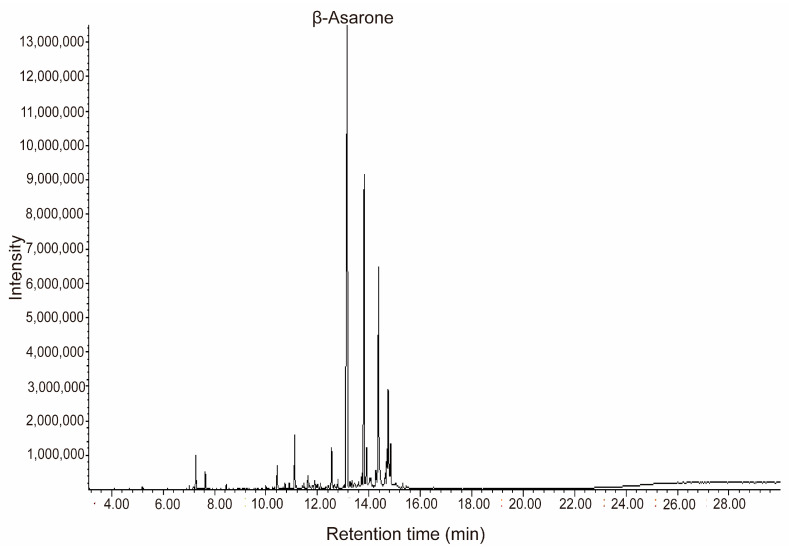
Total ion flow diagram of essential oil.

Through the systematic optimization of single-factor experiments (Figure S1), we established the optimal extraction parameters, including a solid-to-liquid ratio of 1:12, an ultrasonic duration of 55 min, an extraction time of 5.5 h, and a sodium chloride concentration of 4%, achieving an extraction yield of 1.88 ± 0.02% (Figure 2). Variance analysis confirmed the statistical significance of these parameters (Table S1). GC-MS analysis identified 24 chemical components, accounting for 79.26% of the total essential oil content (Figure 3), with β-asarone being the predominant constituent at 55.75% (Table S2).

### 2.2. Pharmacodynamic Evaluation in CUMS-Induced Anxiety Model

#### 2.2.1. Behavioral Assessments

**Figure 4:**
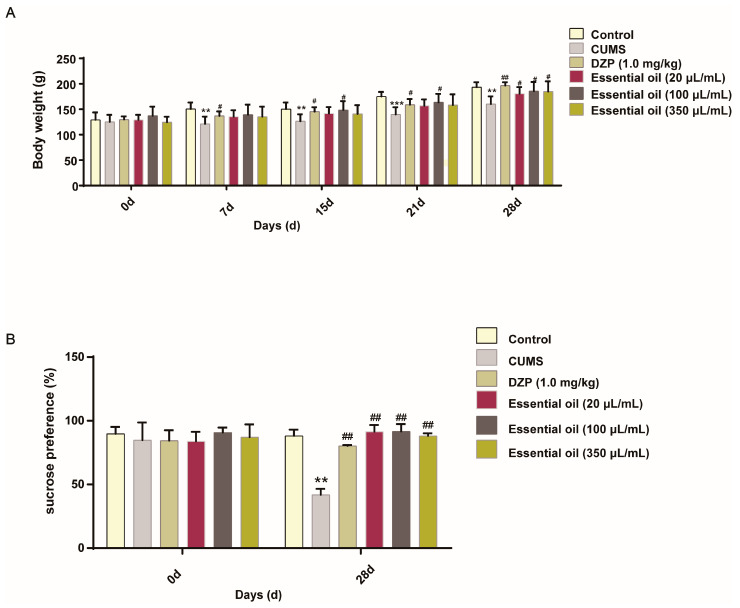
Effect of essential oil (EO) on the chronic unpredictable mild stress (CUMS) model. (**A**) Change in body weight. (**B**) SPT effects in CUMS rats. Data are represented as the mean ± SD (*n* = 6). ** *p* < 0.01, *** *p* < 0.001 vs. the control group, ^#^ *p* < 0.05, ^##^ *p* < 0.01, vs. the model group.

The chronic unpredictable mild stress (CUMS) model was successfully established, as evidenced by a significantly reduced body weight in the model group compared to controls at day 7 (*p* < 0.05, Figure 4A), while treatment with diazepam (DZP, a positive control drug) reversed this effect. The sucrose preference test demonstrated that essential oil (EO) inhalation significantly improved anhedonia in CUMS rats (*p* < 0.05, Figure 4B).

**Figure 5:**
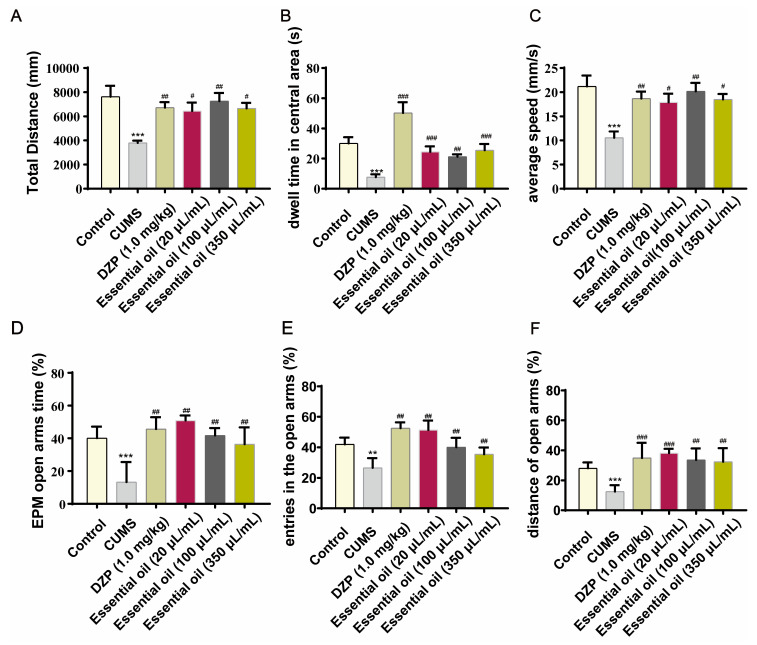
Antianxiety effect of essential oil (EO) inhalation on rats assessed using the OF test (**A**–**C**) and the EPM test (**D**–**F**). Data are presented as the mean ± SD (*n* = 6). ** *p* < 0.01, *** *p* < 0.001 vs. the control group, ^#^ *p* < 0.05, ^##^ *p* < 0.01, ^###^ *p* < 0.001 vs. the model group.

In the open field test (Figure 5A–C), both the EO and DZP groups showed significantly increased central zone duration, total distance traveled, and average speed compared to the model group (*p* < 0.05). The elevated plus maze results (Figure 5D–F) further confirmed the anxiolytic effects, with EO treatment significantly enhancing open arm exploration time and distance (*p* < 0.05; see Supplementary Figure S2).

#### 2.2.2. Safety Evaluation

**Figure 6:**
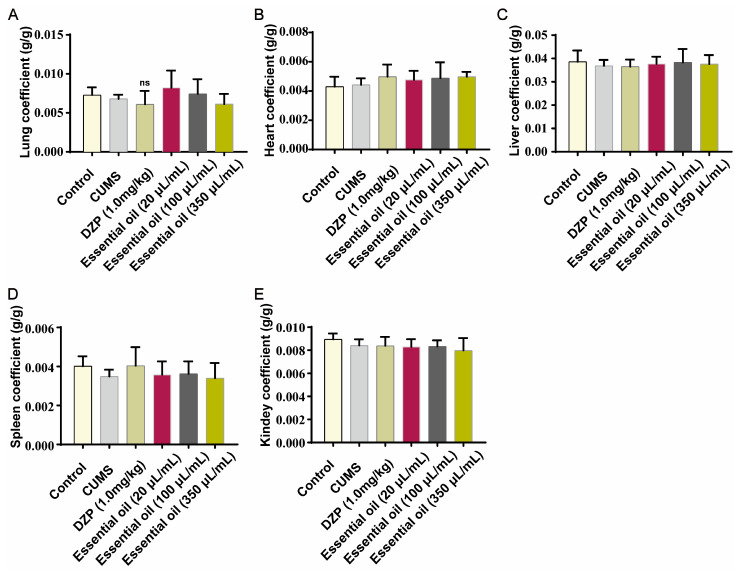
Effect of essential oil (EO) on organ coefficients of rats: (**A**) lung coefficient, (**B**) heart coefficient, (**C**) liver coefficient, (**D**) spleen coefficient, (**E**) kidney coefficient. Data are presented as the mean ± SD (*n* = 4/group). ns indicates no significant difference.

Histopathological examination revealed no significant abnormalities in major organs across all groups (Figure S3A). Organ coefficients showed no statistical differences (*p* > 0.05, Figure 6A–E). Nissl staining demonstrated that EO treatment effectively prevented CUMS-induced hippocampal neuronal damage and the dissolution of Nissl bodies (Figure S3B).

#### 2.2.3. Neurotransmitter Modulation

**Figure 7:**
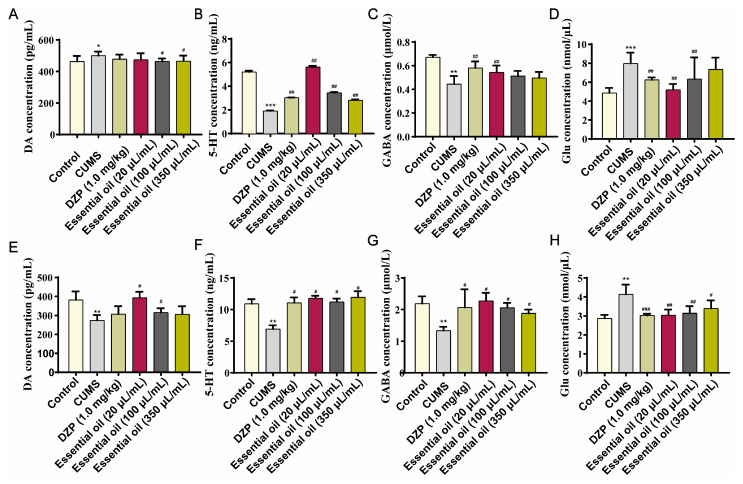
Effects of essential oil (EO) on neurotransmitter content in serum (**A**–**D**) and hippocampal tissue (**E**–**H**) of rats: (**A**,**E**) dopamine (DA), (**B**,**F**) serotonin (5-HT), (**C**,**G**) gamma-aminobutyric acid (GABA), (**D**,**H**) glutamate (Glu). Data are presented as the mean ± SD (*n* = 6/group). * *p* < 0.05, ** *p* < 0.01, *** *p* < 0.001 vs. the control group, ^#^ *p* < 0.05, ^##^ *p* < 0.01, ^###^ *p* < 0.001 vs. the model group, ns indicates no significant difference.

Neurochemical analysis (Figure 7E–H) revealed that medium and low EO concentrations (20, 100 μL/mL) significantly increased hippocampal dopamine (DA) levels (*p* < 0.05). All treatment groups significantly upregulated 5-hydroxytryptamine (5-HT) and γ-aminobutyric acid (GABA) while downregulating glutamate (GLU) content (*p* < 0.05) in both serum (Figure 7A–C) and hippocampal tissue (Figure 7E–H), suggesting modulation of monoamine and amino acid neurotransmitter systems.

### 2.3. Network Pharmacological Analysis

**Figure 8:**
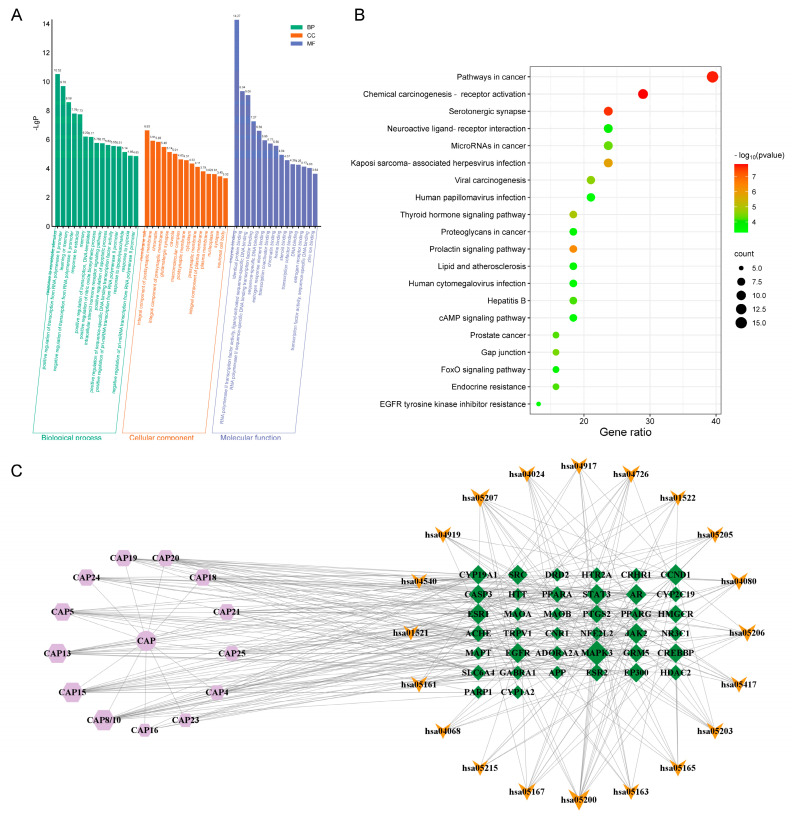
Research on network pharmacology of CAP in treating anxiety. (**A**) GO function enrichment results of essential oil in the treatment of anxiety disorder. (**B**) KEGG enrichment bubble diagram. (**C**) H−C−T−P network diagram. Circles represent traditional Chinese medicine; hexagons represent components; diamonds represent targets; V represents pathways.

**Figure 9:**
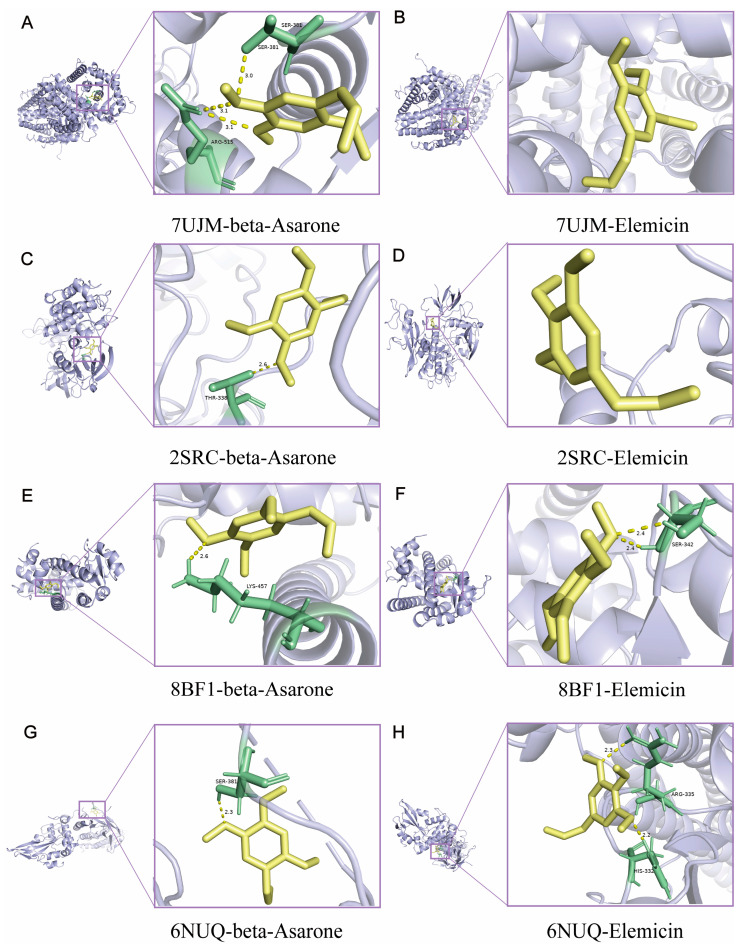
Molecular docking diagrams of chemical compositions to targets: (**A**) ESR1–beta-asarone; (**B**) ESR1–elemicin; (**C**) SRC–beta-asarone; (**D**) SRC–elemicin; (**E**) PPARG–beta-asarone; (**F**) PPARG–elemicin; (**G**) STAT3–beta-asarone; (**H**) STAT3–elemicin.

Cytoscape network analysis (3.10.0) identified 25 major active components (Figure S4), with β-asarone (degree = 51) and elemicin (degree = 50) showing the highest connectivity (Table S5). Intersection analysis of 295 predicted targets (from SwissTargetPrediction), with 2706 anxiety-related targets (from OMIM/DisGeNET/GeneCards), yielded 135 key targets (Figure 8, Table S7). Molecular docking confirmed strong binding (binding energy < −5.0 kcal/mol) between core targets (ESR1, SRC, PPARG) and active compounds (Tables S6 and S8, Figure 9), except for the STAT3–elemicin interaction.

### 2.4. Preparation and Characterization of Essential Oil Microemulsion

#### 2.4.1. Formulation Optimization

**Figure 10:**
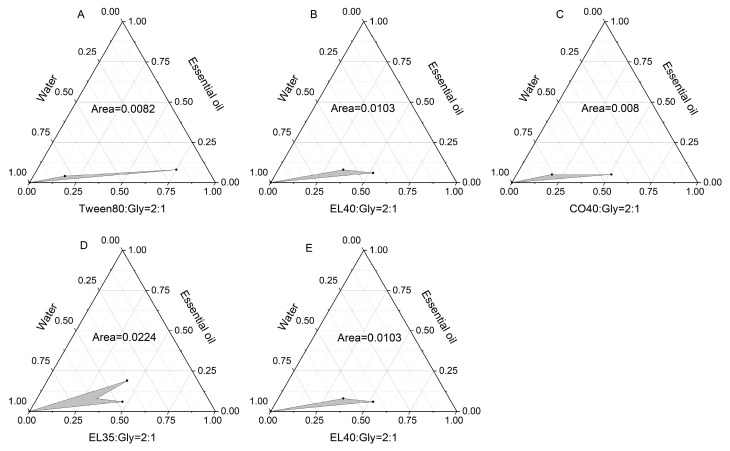
Pseudo-ternary phase diagrams of different surfactant–essential oil–water systems. (**A**) Tween 80; (**B**) EL 40; (**C**) CO 40; (**D**) EL 35; (**E**) EL40.

**Figure 11:**
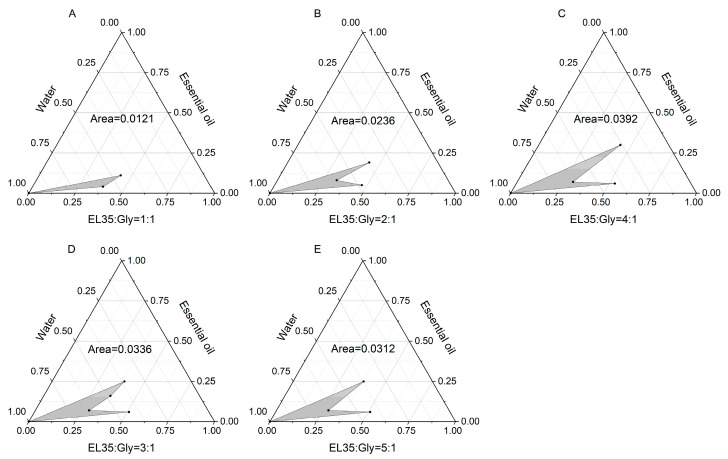
Pseudo-ternary phase diagrams of essential oil microemulsions with different Km values. (**A**) EL 35: Gly = 1:1; (**B**) EL 35: Gly = 2:1; (**C**) EL 35: Gly = 4:1; (**D**) EL 35: Gly = 3:1; (**E**) EL 35: Gly = 5:1.

Pseudo-ternary phase diagrams (Figure 10 and Figure 11) demonstrated the maximal microemulsion region at Km = 4:1 (surfactant: cosurfactant ratio, Figure 11 and Figure S5), which was selected for preparation.

#### 2.4.2. Physicochemical Properties

**Figure 12:**
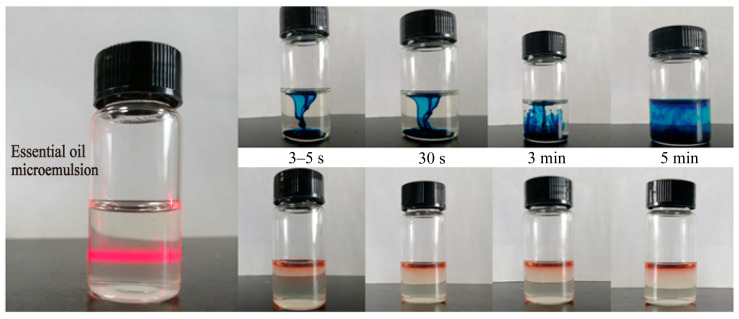
Essential oil microemulsion type identification diagram.

**Figure 13:**
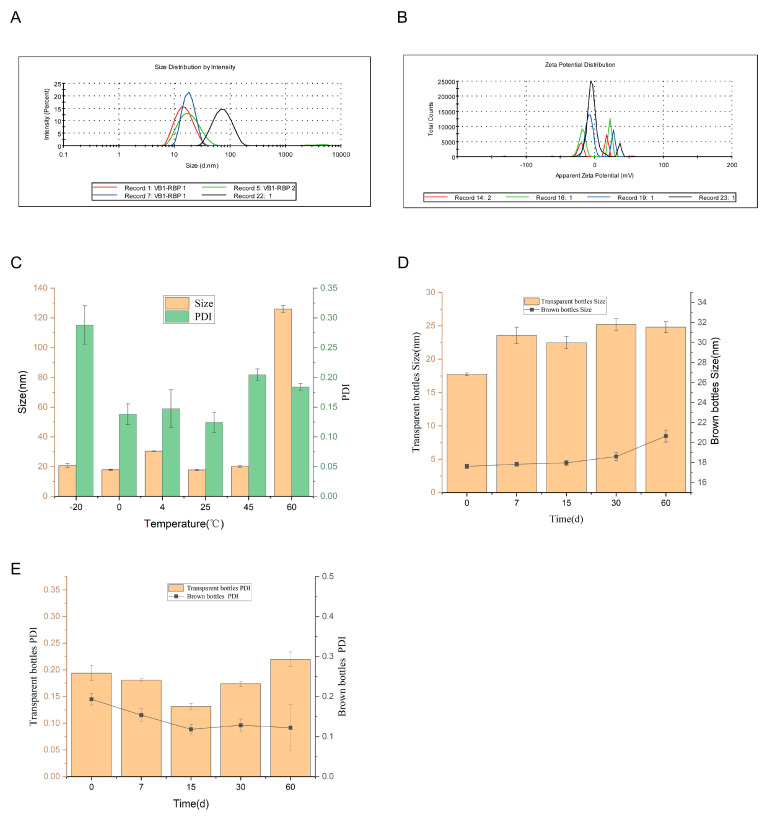
Characterization and stability of prepared essential oil microemulsion. (**A**) Essential oil microemulsion particle size distribution. (**B**) Essential oil microemulsion zeta potential. (**C**) Particle size of essential oil microemulsion at different temperatures. (**D**) Particle size of essential oil microemulsion over different days. (**E**) PDI of essential oil microemulsion over different days.

As shown in Figure 12, the optimized microemulsion appears as a pale yellow transparent liquid (Table S4) with good fluidity (pH 5.35). Under parallel light irradiation, it exhibits the Tyndall effect. When the water-soluble dye methylene blue and the oil-soluble dye Sudan III are added to the microemulsion system, it can be observed that methylene blue immediately diffuses throughout the microemulsion, while Sudan III almost floats on the surface of the microemulsion. The dye diffusion test confirmed the O/W type (Figure 12). Laser particle size analysis showed a uniform distribution with a mean diameter of 17.74 ± 0.55 nm, a PDI of 0.065 ± 0.033, and a zeta potential of −0.452 ± 0.308 mV (Figure 13A,B, Table S3).

#### 2.4.3. Stability Studies

Centrifugation tests (Table 1) and long-term stability evaluations (Figure 13C–E) confirmed excellent physical stability, with no significant changes in key parameters after 60 days of storage at room temperature.

## 3. Methods and Materials

### 3.1. Chemicals and Reagents

Acorus calamus, sandalwood, peppermint, and orange peel were obtained from Anhui Xusong Traditional Chinese Medicine Decoction Pieces Co., Ltd. (Bozhou, Anhui, China). Diazepam injection (10 mg/2 mL) was provided by the Wuhan Mental Health Center. Paraformaldehyde (4%) and ELISA kits for mouse 5-HT, Glu, and γ-GABA were purchased from Jianglai Biological Co., Ltd. (Shanghai, China). All reagents were of analytical grade.

### 3.2. Animals

Male Sprague–Dawley (SD) rats (5 weeks old, 120 ± 10 g) were purchased from the Hubei Provincial Laboratory Animal Research Center. The experimental protocol was approved by the Animal Ethics Committee of Wuhan Polytechnic University (License No.: WPO202208001). The animals were housed under specific pathogen-free (SPF) conditions at 23 ± 1 °C with a 12 h light/dark cycle and had free access to food and water. All procedures strictly followed the Chinese national guidelines for the ethical use of laboratory animals.

### 3.3. Equipment

The following instruments were used: whole-body inhalation exposure system (Shanghai Yuyan Instruments Co., Ltd., Shanghai, China), elevated plus maze and SuperMaze animal behavior analysis system (Shanghai Xinruan Information Technology Co., Ltd., Shanghai, China), spontaneous locomotor activity test system and DigBehv behavior analysis system (Shanghai Jiliang Software Technology Co., Ltd., Shanghai, China), refrigerated high-throughput tissue homogenizer (Ningbo Xinzhi Biotechnology Co., Ltd., Ningbo, China), gas chromatography–mass spectrometer (GC-MS, Agilent 7890A/5975C, Los Angeles, CA, USA), and Malvern particle size analyzer (ZEN-3600, Malvern Instruments, Malvern, UK).

### 3.4. Preparation of Essential Oil

Powdered herbs (Acorus calamus, sandalwood, orange peel, and peppermint in a 3:2:1:1 ratio, totaling 110 g) were subjected to steam distillation with ultrasonic assistance. The oil–water mixture was extracted with petroleum ether and concentrated using a rotary evaporator. Single-factor experiments and response surface methodology were employed to optimize the extraction parameters (solid-to-liquid ratio, ultrasonic time, extraction duration, and NaCl concentration).

### 3.5. GC-MS Analysis

Essential oil components were analyzed using an HP-5MS column (30 m × 250 μm × 0.25 μm) under the following conditions: injector temperature 250 °C; split ratio 20:1; helium flow rate 1 mg/min. The temperature program was as follows: 40 °C (1 min) → 120 °C at 4 °C/min → 150 °C at 2 °C/min (3 min) → 220 °C at 5 °C/min → 300 °C at 20 °C/min (1 min). Mass spectrometry parameters included: ion source temperature 230 °C; interface temperature 301 °C; scan range *m*/*z* 20–500; solvent delay 3 min. Components were identified by comparing mass spectra with the NIST11.L library.

### 3.6. CUMS Model Establishment

After 1 week of acclimatization, rats were randomly divided into six groups (*n* = 10/group): normal control (no stress/treatment), CUMS model (stress only), positive control (diazepam, DZP, 1.0 mg/kg, i.p.), and three EO dose groups (20, 100, and 350 μL/mL, inhalation). The 4-week experimental protocol included: 12 h light/dark reversal twice weekly, 24 h food/water deprivation once weekly, 5 min acetic acid stimulation (0.3%) once weekly, and 45° cage tilting for 12 h twice weekly, with additional stressors rotated daily. The EO groups received 30 min of inhalation treatment daily, followed by stressor application 30 min later. The positive control DZP was administered 15 min before stress exposure. All behavioral tests were conducted 24 h after the final treatment.

### 3.7. Organ Coefficient and Histopathology

Following euthanasia, heart, liver, spleen, lung, kidney, and hippocampal tissues were dissected. Organ coefficients were calculated as (organ weight/body weight) × 100%. Tissues were fixed in 4% paraformaldehyde, processed for H&E and Nissl staining, and examined under light microscopy. For histological analyses, 4 animals per group were randomly selected (*n* = 4/group).

### 3.8. Behavioral Evaluation

#### 3.8.1. Sucrose Preference Test (SPT)

The sucrose preference test (SPT) was conducted on day 27 of treatment following a 48 h acclimatization period, during which rats were exposed to two drinking bottles (one containing a 1% sucrose solution and the other containing water). The positions of the bottles were alternated every 12 h to eliminate side preference, and animals showing <60% sucrose preference (*n* = 2) were excluded. After 14–18 h of food and water deprivation, rats were simultaneously presented with two pre-weighed bottles (300 mL Pyrex) containing either 1% sucrose solution or ultrapure water for 6 h under controlled conditions (23 ± 1 °C, 55 ± 5% humidity), with bottle positions counterbalanced relative to the final acclimatization position.

$$\text{Sucrose preference (\%)}=\frac{\text{sucrose consumption}}{\text{sucrose solution}+\text{water consumption}}\times 100$$

#### 3.8.2. Open Field Test (OFT)

The open field test was conducted in a square arena (50 × 50 × 40 cm), with the floor divided into 16 equal grids (12.5 × 12.5 cm each), where the central 4 grids (25 × 25 cm) were designated as the center zone. Following a 10 min room acclimation period, individual rats were placed in the center of the arena, and their spontaneous activity was video recorded for 6 min (30 fps) under standardized conditions (50 lux illumination, <55 dB background noise). Behavioral parameters, including movement trajectory, total distance traveled, center zone duration, and average velocity, were automatically analyzed using DigBehv software. Between trials, the arena was thoroughly cleaned with 75% ethanol and lint-free wipes to eliminate odor cues, and all testing was performed by experimenters blinded to the treatment groups.

#### 3.8.3. Elevated Plus Maze (EPM)

The elevated plus maze test was conducted using a plus-shaped apparatus (+) consisting of two open arms (50 × 10 × 0.6 cm with edge rims) and two enclosed arms (50 × 10 × 40 cm with walls) extending from a central platform (10 × 10 cm), elevated 70 cm above the floor. Following 60 s of habituation on the central platform, rats were allowed to freely explore the maze for 6 min under standardized lighting (30 lux). Behavioral parameters, including time spent in open/closed arms and the number of arm entries (defined as all four paws in an arm), were automatically tracked using EthoVision XT 15 (30 fps) and manually verified by blinded observers. The maze was constructed of gray PVC with non-reflective surfaces and was cleaned with 70% ethanol between trials to eliminate odor cues. Anxiety-like behavior was quantified using the open arm time ratio [open/(open + closed) time] and the entry ratio [open/(open + closed) entries], with typical control values ranging from 30 to 40% for open arm time.

### 3.9. Neurotransmitter Measurement

Blood samples were centrifuged (3000 rpm for 10 min at 4 °C) to obtain serum. Hippocampal tissues were homogenized in PBS (1:10), then centrifuged (5000 rpm for 5–10 min), and the supernatants were analyzed for 5-HT, DA, Glu, and γ-GABA using commercial ELISA kits. All remaining animals (*n* = 6/group) were used for hippocampal homogenate experiments to ensure adequate statistical power for neurochemical analyses.

### 3.10. Network Pharmacology

Bioactive compounds were screened using the TCMSP (https://www.tcmsp-e.com/index.php; accessed on 15 December 2024) and PubChem databases (http://pubchem.ncbi.nlm.nih.gov; accessed on 15 December 2024) [37,38]. Target prediction was performed using SwissTargetPrediction (http://swisstargetprediction.ch/; accessed on 15 December 2024). Anxiety-related targets were retrieved from DisGeNET (https://www.disgenet.org/; accessed on 15 December 2024), OMIM (https://omim.org/search; accessed on 15 December 2024), DrugBank (https://go.drugbank.com/; accessed on 15 December 2024), and GeneCards (https://www.genecards.org/; accessed on 16 December 2024). A compound-target-pathway network was constructed using Cytoscape 3.9.1, and KEGG pathway analysis was conducted via Bioinformatics (accessed on 16 December 2024).

### 3.11. Molecular Docking

Protein structures were downloaded from PDB (https://www.rcsb.org/), prepared using PyMOL, and docked with ligands (β-asarone and elemicin) via AutoDock Vina 1.1.2. Binding energies < −5.0 kcal/mol indicated strong interactions. Results were visualized with PyMOL 2.5.0.

### 3.12. Microemulsion Preparation

Optimal microemulsions were prepared using EL35 and glycerol (Km = 4:1) as surfactants. Pseudo-ternary phase diagrams were constructed to identify microemulsion regions. Formulations were characterized for particle size, PDI, zeta potential, and stability.

### 3.13. Drawing of Pseudo Ternary Phase Diagram

A pseudo-ternary phase diagram is a simplified phase diagram used to describe the phase behavior of components in complex systems. It is usually used in systems with four or more components. By combining two or more components into a “pseudo component”, the high-dimensional phase diagram is simplified to the form of a ternary phase diagram. The mixed surfactant, oil phase, and water were taken as the vertices of the pseudo-ternary phase diagram to explore the effects of different surfactants, different cosurfactants, and different Km values on the formation of microemulsion. The ternary phase diagram was created using Origin 2018 software to determine the microemulsion area and calculate its size.

### 3.14. Statistical Analysis

Data were analyzed using GraphPad Prism 8.0 (mean ± SD). One-way ANOVA followed by post hoc tests was applied. *p* < 0.05 was considered statistically significant.

## 4. Discussion

The present study provides compelling evidence for the anxiolytic effects of essential oil (EO) through a comprehensive investigation that combines phytochemical analysis, behavioral pharmacology, neurochemical assessment, and network pharmacology approaches. Our findings demonstrate that EO inhalation effectively ameliorates anxiety-like behaviors in CUMS model rats, possibly through the modulation of monoamine neurotransmitter systems and GABAergic signaling pathways.

The GC-MS analysis identified 24 bioactive components in EO, with β-asarone (55.75%) as the predominant constituent. This phytochemical profile is particularly noteworthy, as β-asarone has been previously reported to possess neuroprotective and anxiolytic properties through GABA receptor modulation [39]. The successful standardization of extraction parameters (1:12 solid–liquid ratio, 55 min ultrasonication, 5.5 h extraction time, 4% NaCl), yielding 1.88% essential oil, provides a reproducible method for future pharmacological studies and potential clinical applications.

Mounting evidence indicates that administration frequency critically determines the therapeutic outcomes of plant EOs. Our experimental protocol, which employed daily 30 min inhalation sessions over 4 weeks, achieved a significant reversal of CUMS-induced behavioral deficits. This finding is strongly supported by pharmacokinetic studies demonstrating the cumulative effects of EO components [40]. Behavioral assessments revealed that EO administration significantly reversed CUMS-induced abnormalities across multiple paradigms. The restoration of sucrose preference suggests antidepressant-like activity, while increased open-arm exploration in the EPM and central zone activity in the OFT demonstrate robust anxiolytic effects comparable to diazepam. These findings align with previous reports on the mood-regulating properties of individual components (*Acorus calamus*, *Santalum album*, *Citrus limon*, and *Mentha piperita*) while highlighting the potential synergistic benefits of the compound formulation [28,30,41,42,43].

At the neurochemical level, EO treatment normalized CUMS-induced disturbances in hippocampal neurotransmitter levels. The observed increases in 5-HT and GABA, along with decreased glutamate, suggest a multimodal mechanism of action. Particularly interesting is the dose-dependent elevation of DA by low and medium EO concentrations, as dopaminergic signaling in the mesolimbic pathway plays a crucial role in reward processing and motivation—key domains affected by anxiety and depression [44,45]. The restoration of Nissl body integrity in hippocampal neurons further supports the neuroprotective potential of EO against stress-induced neuronal damage.

Network pharmacology and molecular docking analyses provide important insights into the polypharmacology of EO. The identification of ESR1, SRC, PPARG, and STAT3 as core targets suggests their involvement in neuroendocrine regulation, neuroinflammation, and synaptic plasticity. Pathway enrichment analysis implicates cAMP signaling and neuroactive ligand–receptor interactions as potential mediators of EO’s effects, consistent with the known mechanisms of conventional anxiolytics [46,47,48]. The strong binding affinity (≤−5.0 kcal/mol) between β-asarone/elemicin and these targets provides molecular-level validation for our network predictions.

The development of a stable O/W microemulsion (17.74 nm particle size, PDI 0.065, −0.452 mV zeta potential) addresses critical formulation challenges associated with essential oils, including volatility and oxidative instability. The excellent centrifugal and long-term stability (60 days at room temperature) of this preparation enhances its potential for clinical translation.

## 5. Conclusions

In summary, this study systematically demonstrated that EO microemulsion exhibits significant anxiolytic effects in CUMS model rats through multimodal modulation of monoaminergic and GABAergic neurotransmission. By integrating behavioral, neurochemical, and network pharmacological approaches, it established a new paradigm for investigating the pharmacological basis of traditional aromatherapy. The successful combination of traditional medicine with modern formulation technology provides an important reference for the future development and application of compound aromatic essential oils with anxiolytic properties.

## Figures and Tables

**Table 1:** Changes in particle size, zeta, and polydispersity index (PDI) of microemulsions at different centrifugal rates.

	Precentrifugation			Postcentrifugation		
Centrifugal Rate (rpm/min)	Particle Size (nm)	Zeta Potential (mv)	PDI	Particle Size (nm)	Zeta Potential (mv)	PDI
2000	20.73 ± 0.14	−0.77	0.16 ± 0.015	18.55 ± 0.64	−0.67	0.16 ± 0.022
4000	20.47 ± 0.18	−1.31	0.21 ± 0.0045	18.26 ± 0.15	−0.99	0.20 ± 0.012
6000	20.09 ± 0.23	−0.99	0.210	17.63 ± 0.19	−0.95	0.15 ± 0.0085
8000	19.32 ± 0.25	−1.21	0.17 ± 0.023	17.47 ± 0.095	−0.91	0.15 ± 0.0035
10,000	22.43 ± 0.085	−1.27	0.22 ± 0.014	17.88 ± 0.36	−0.83	0.15 ± 0.029

## Data Availability

Data are contained within the article.

## Supplementary Materials

The following supporting information can be downloaded at: https://www.mdpi.com/article/10.3390/molecules30122652/s1, Figure S1: The influence of different factors on Essential oil was analyzed by single factor analysis; Figure S2: EPM experiment trajectory of different groups of CUMS rats; Figure S3: Effect of Essential Oil on CUMS rat tissue; Figure S4: Network Pharmacology Investigation of Essential oil in the treatment of Anxiety; Figure S5. Pseudo-ternary phase diagrams of different cosurfactant -Essential oil - water system; Table S1: Variance significance analysis; Table S2: Analysis results of GC-MS detection on Essential Oil; Table S3: Particle Size, PDI, and Potential of Essential Oil microemulsion; Table S4: Viscosity, conductivity, and pH of Essential Oil microemulsion; Table S5: Chemical profile of the principal active ingredients; Table S6: Core target information table; Table S7: KEGG pathway enrichment results; Table S8: Docking results of target protein and active compound.

## Abbreviations

EO, essential oil; Gly, glycerol; PG, propylene glycol; EA, ethanol absolute; GC-MS, gas chromatography–mass spectrometry; SPT, sucrose preference test; OFT, open field test; EPM, elevated plus maze; CUMS, chronic unpredictable mild stress; H & E, hematoxylin and eosin; PPI, protein–protein interaction; H-C-T-P, herb–component–target–pathway; γ-GABA, gamma amino butyric acid; 5-HT, 5-hydroxytryptamine; DA, dopamine; GLU, glutamate, PDI: polydispersity index. DZP: diazepam.
