# Supplementary material for: Anti-Anxiety Effects of Essential Oil Microemulsion in Chronic Unpredictable Mild Stress-Induced Rats: Preparation, Characterization, and Mechanisms
Source: Molecules. 2025 Jun 19;30(12):2652. doi: 10.3390/molecules30122652 (PMC12196394; doi:10.3390/molecules30122652)
Supplement: Supplementary file 1 [file molecules-30-02652-s001.zip › molecules-3493467-supplementary.pdf]

**Supplementary data**

# Anti-Anxiety Effects of Essential Oil Microemulsion in Chronic Unpredictable Mild Stress-induced Rats: Comprehensive Study on Preparation, Characterization, Stability and Neurobiological Mechanisms

Wenxia Tang [1], Pan Jiang[1], Ke Hu[1], Duo Mei, Qinghao Jiao, Yan Li, Yanping Deng, Jun Wang, Ran Gao, Xin Chen[*], Jie Yu[*]

School of Life Science and Technology, Wuhan Polytechnic University, Wuhan, 430023, China;

tangwenxia2022@163.com (W.T.); 15906803974@163.com (P.J.); huke2077@163.com (K.H.); 18672323259@163.com (D.M.); 18899686667@163.com (Q.J.); 17764061495@163.com (Y.L.); dengyanping20@126.com (Y.D.); wangjun202502@126.com (J.W.); h2025126@126.com (R.G.)

Correspondence: yujie0326@163.com (J.Y.); chenxin_0001@126.com (X.C.)

[1]These authors contributed equally

**Keywords:** Herbal, Microemulsion**,** Aromatic plant essential oil, Anxiety, Sedative and hypnotic effects, Network pharmacology

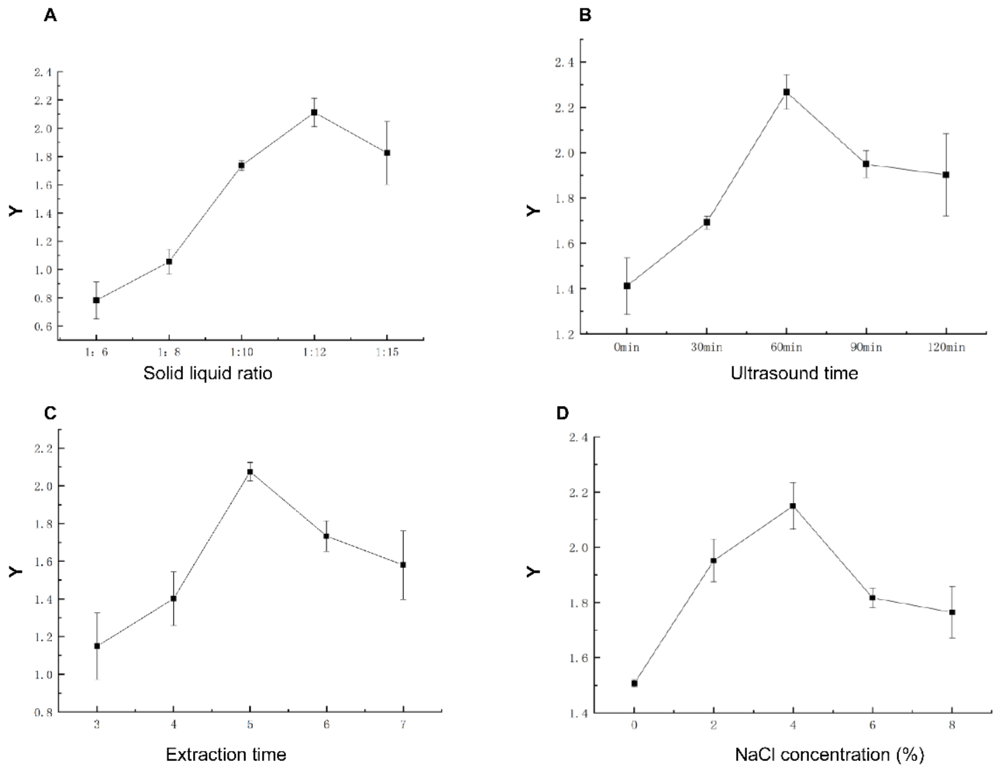


**Figure S1.** The influence of different factors on Essential oil was analyzed by single factor analysis. (A) Solid liquid ratio. (B) Ultrasound time. (C) Extraction time. (D) NaCl concentration.

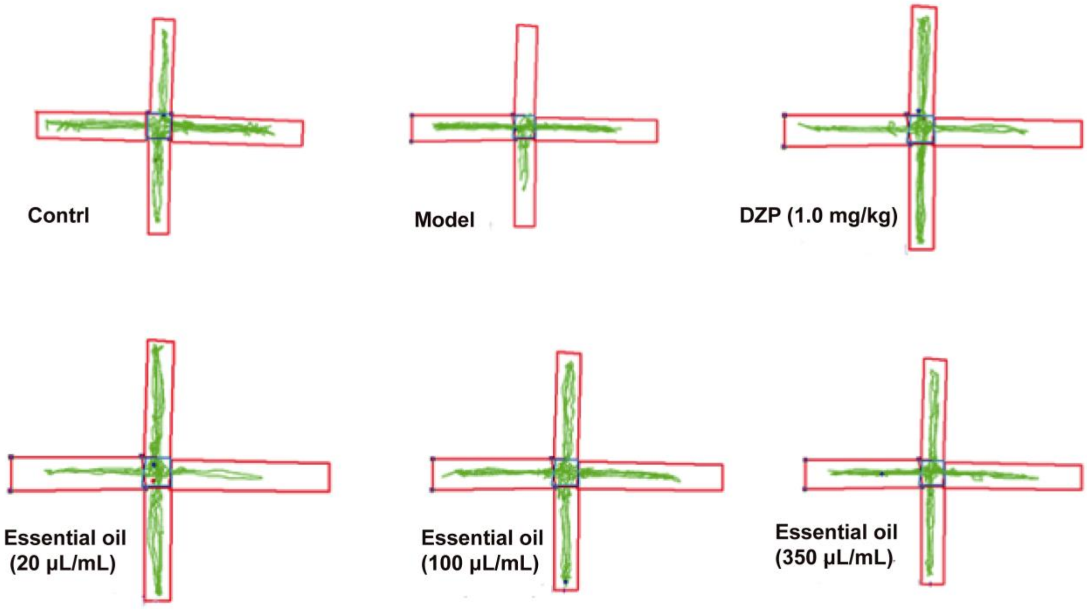


**Figure S2.** EPM experiment trajectory of different groups of CUMS rats.

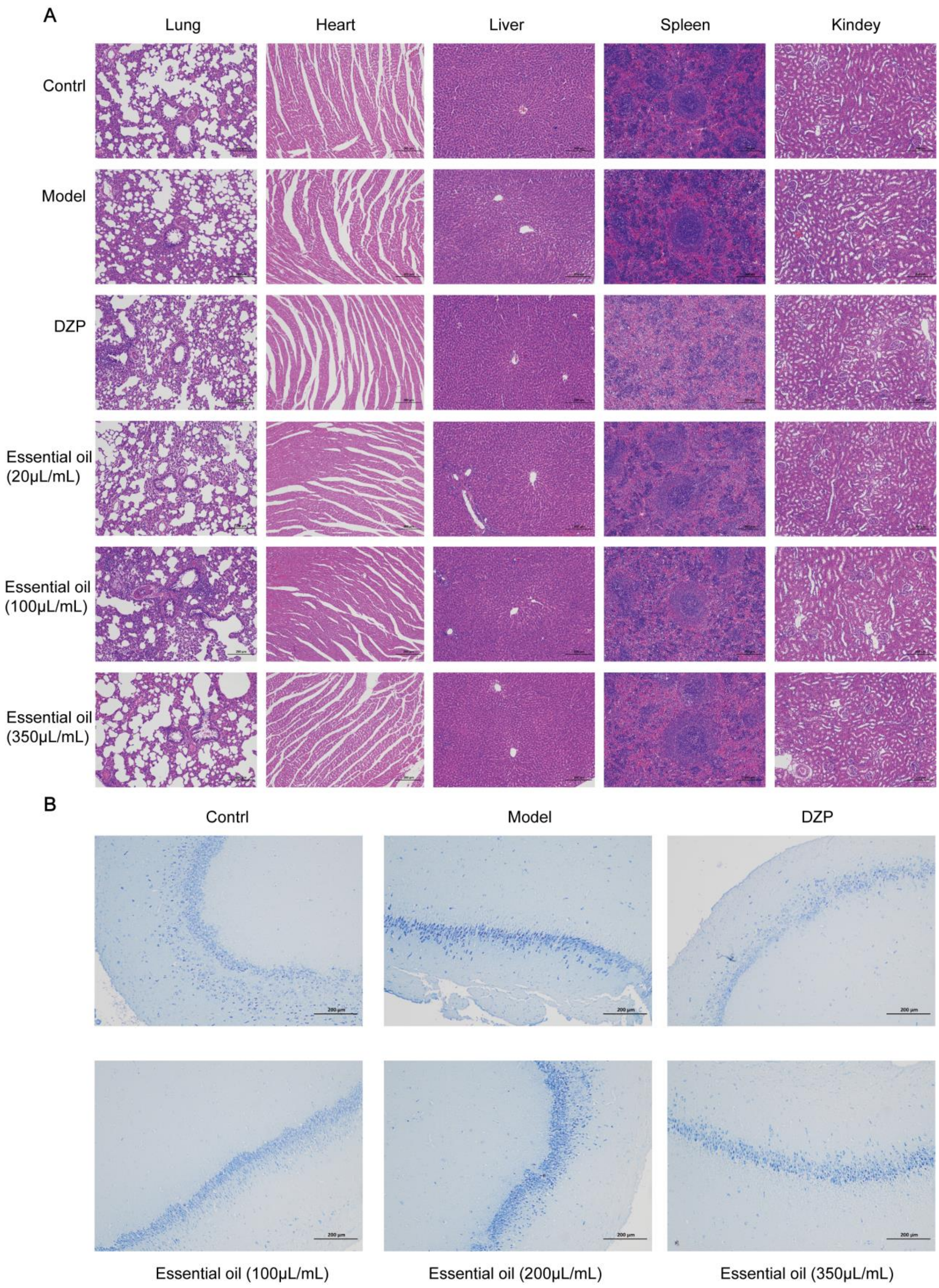


**Figure S3.** Effect of Essential Oil on CUMS rat tissue. A: H & E staining results of f Essential Oil on tissues and organs of CUMS rats. B: Results of Nissl staining of hippocampal tissues of Essential Oil on CUMS rats. (Bar=200μm).

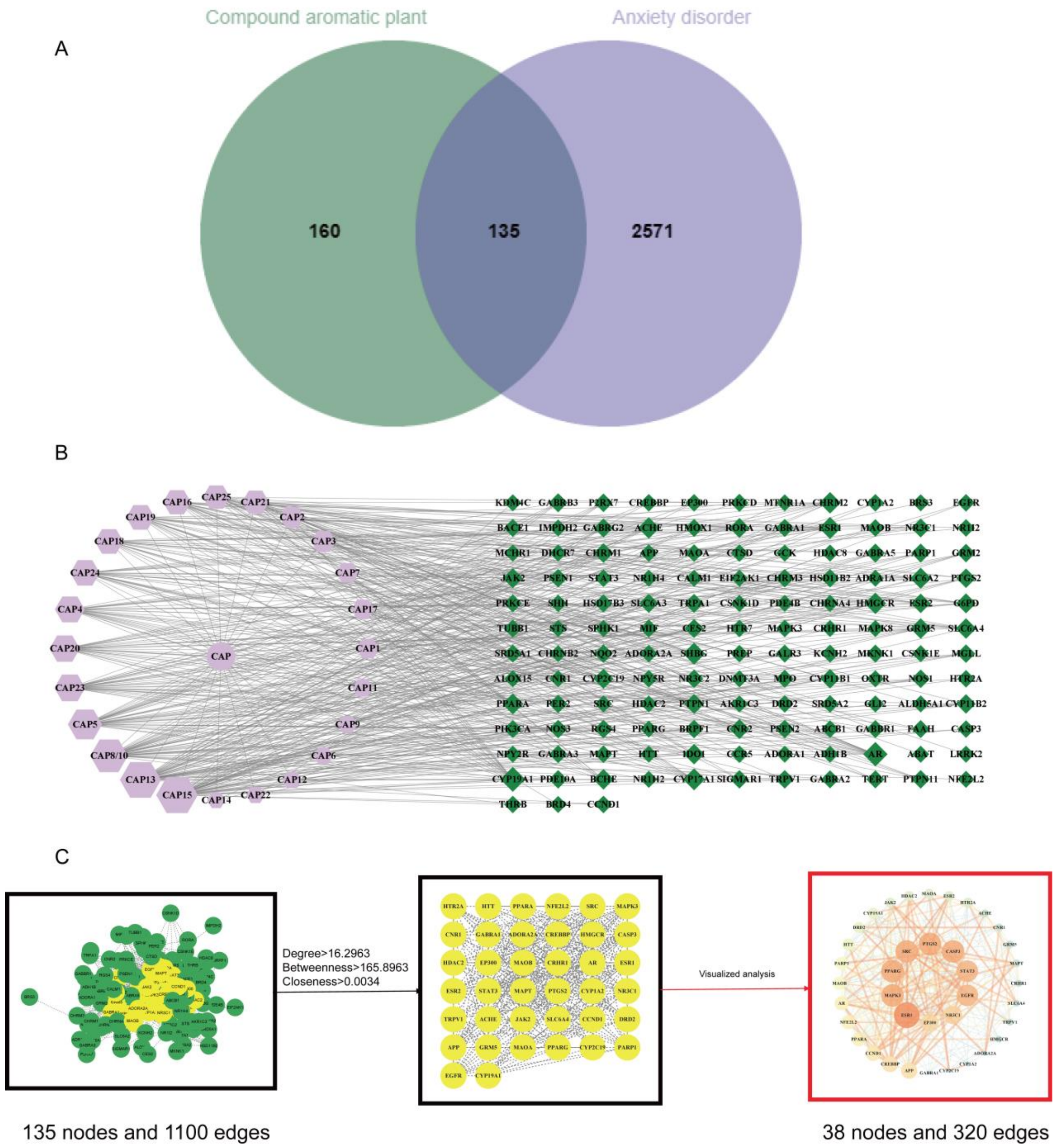


**Figure S4.** Network Pharmacology Investigation of Essential oil in the treatment of Anxiety. (A) Disease-drug target Venn diagram. (B) Composition-target network of Compound aromatic plant. Circles are for traditional Chinese medicine; hexagon are component； diamonds are target. (C) Core target PPI network.

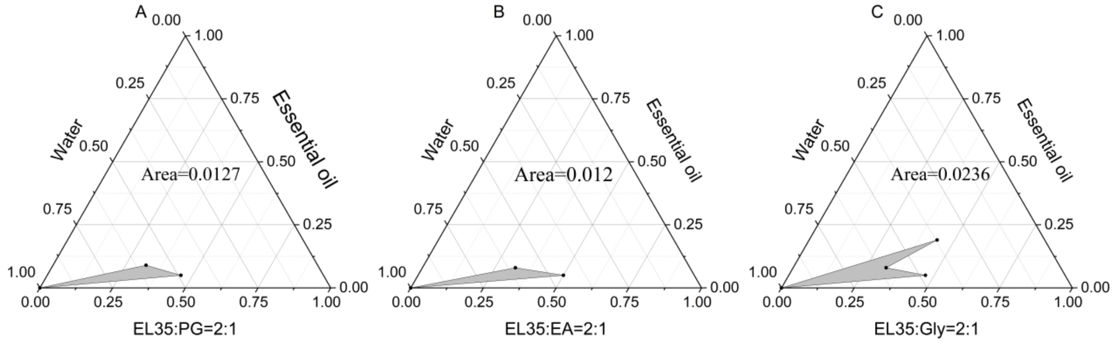


**Figure S5.** Pseudo-ternary phase diagrams of different cosurfactant -Essential oil - water system

**Table S1:** Variance significance analysis.

| Source | Sum of Squares | df | Mean Square | F-value | p-value | |
|---|---|---|---|---|---|---|
| **Model** | 0.6721 | 14 | 0.0480 | 28.08 | < 0.0001 | significant |
| A- Solid liquid ratio | 0.0441 | 1 | 0.0441 | 25.80 | 0.0002 | |
| B- Ultrasound time | 0.0079 | 1 | 0.0079 | 4.64 | 0.0492 | |
| C- Extraction time | 0.2210 | 1 | 0.2210 | 129.29 | < 0.0001 | |
| D-Nacl concentration | 0.0500 | 1 | 0.0500 | 29.23 | < 0.0001 | |
| AB | 0.0162 | 1 | 0.0162 | 9.46 | 0.0082 | |
| AC | 0.0072 | 1 | 0.0072 | 4.24 | 0.0587 | |
| AD | 0.0009 | 1 | 0.0009 | 0.5174 | 0.4838 | |
| BC | 0.0027 | 1 | 0.0027 | 1.58 | 0.2300 | |
| BD | 0.0199 | 1 | 0.0199 | 11.65 | 0.0042 | |
| CD | 0.0357 | 1 | 0.0357 | 20.90 | 0.0004 | |
| $A^2$ | 0.1875 | 1 | 0.1875 | 109.71 | < 0.0001 | |
| $B^2$ | 0.0326 | 1 | 0.0326 | 19.05 | 0.0006 | |
| $C^2$ | 0.0873 | 1 | 0.0873 | 51.06 | < 0.0001 | |
| $D^2$ | 0.0554 | 1 | 0.0554 | 32.39 | < 0.0001 | |
| **Residual** | 0.0239 | 14 | 0.0017 | | | |
| Lack of Fit | 0.0180 | 10 | 0.0018 | 1.21 | 0.4639 | not significant |
| Pure Error | 0.0060 | 4 | 0.0015 | | | |
| **Cor Total** | 0.6960 | 28 | | | | |

Note: P<0.01 means significant, P<0.001 means extremely significant.

**Table S2:** Analysis results of GC-MS detection on Essential Oil.

| No. | Retention Time（min） | Compound | Molecular Formula | CAS | Relative Amount (%） |
|---|---|---|---|---|---|
| 1 | 7.014 | Menthone | $C_{10}H_{18}O$ | 89-80-5 | 0.15 |
| 2 | 7.274 | Menthol | $C_{10}H_{20}O$ | 15356-70-4 | 1.28 |
| 3 | 7.640 | Estragole | $C_{10}H_{12}O$ | 140-67-0 | 0.75 |
| 4 | 8.458 | Piperitone | $C_{10}H_{16}O$ | 89-81-6 | 0.22 |
| 5 | 10.434 | Methyl eugenol | $C_{11}H_{14}O_2$ | 93-15-2 | 1.09 |
| 6 | 10.741 | Caryophyllene-(I1) | $C_{15}H_{24}$ | 87-44-5 | 0.27 |
| 7 | 10.912 | (+)-Calarene | $C_{15}H_{24}$ | 17334-55-3 | 0.29 |
| 8 | 11.115 | Methyl isoeugenol | $C_{11}H_{14}O_2$ | 93-16-3 | 2.13 |
| 9 | 11.475 | α-Curcumene | $C_{15}H_{22}$ | 644-30-4 | 0.19 |
| 10 | 11.629 | Methyl isoeugenol | $C_{11}H_{14}O_2$ | 93-16-3 | 0.86 |
| 11 | 11.897 | Shyobunone | $C_{15}H_{24}O$ | 21698-44-2 | 0.48 |
| 12 | 12.011 | (+)-Delta-cadinene | $C_{15}H_{24}$ | 483-76-1 | 0.31 |
| 13 | 12.554 | Elemicin | $C_{12}H_{16}O_3$ | 487-11-6 | 2.22 |
| 14 | 12.796 | Caryophyllene oxide | $C_{15}H_{24}O$ | 1139-30-6 | 0.44 |
| 15 | 13.158 | β-Asarone | $C_{12}H_{16}O_3$ | 5273-86-9 | 55.75 |
| 16 | 13.265 | (-)-Perillaldehyde | $C_{10}H_{14}O$ | 2111-75-3 | 0.45 |
| 17 | 13.341 | (+)-Alpha-funebrene | $C_{15}H_{24}$ | 50894-66-1 | 0.40 |
| 18 | 13.441 | Agarospirol | $C_{15}H_{26}O$ | 1460-73-7 | 0.51 |
| 19 | 13.603 | α-Cadinol | $C_{15}H_{26}O$ | 481-34-5 | 0.35 |
| 20 | 13.727 | β-Bisabolol | $C_{15}H_{26}O$ | 374-18-2 | 0.86 |
| 21 | 14.044 | Cedren-13-ol, 8- | $C_{15}H_{24}O$ | 18319-35-2 | 1.06 |
| 22 | 14.091 | Cyclolongifolene oxide | $C_{15}H_{24}$ | 156-11-4 | 0.44 |
| 23 | 14.271 | Farnesol | $C_{15}H_{26}O$ | 4602-84-0 | 1.04 |
| 24 | 14.700 | Bergamotenol | $C_{15}H_{24}O$ | 88034-74-6 | 1.98 |
| 25 | 14.753 | Lanceol, cis | $C_{15}H_{24}O$ | 10067-28-4 | 5.74 |

**Table S3:** Particle Size, PDI, and Potential of Essential Oil microemulsion.

| Mixed surfactant：CAPEOs | Particle Size | PDI | Zeta Potential |
|---|---|---|---|
| 9：1 | 14.30 ± 0.27 | 0.13 ± 0.0091 | -0.19 ± 0.121 |
| 8：2 | 17.16 ± 0.12 | 0.17 ± 0.030 | -0.16 ± 0.012 |
| 7：3 | 17.74 ± 0.55 | 0.065 ± 0.033 | -0.45 ± 0.31 |
| 6：4 | 64.91 ± 0.12 | 0.16 ± 0.0062 | -0.30 ± 0.12 |

**Table S4:** Viscosity, conductivity, and pH of Essential Oil microemulsion.

| Sample | Viscosity (mpa·s) | Conductivity (μs/cm) | PH |
|---|---|---|---|
| CAPEOs microemulsion | 29.50 ± 0.45 | 139.65 ± 1.90 | 5.35 ± 0.25 |

**Table S5:** Chemical profile of the principal active ingredients.

| No. | Molecule name | Molecular Formula | CAS | Degree |
|---|---|---|---|---|
| CAP15 | beta-Asarone | $C_{12}H_{16}O_3$ | 5273-86-9 | 51 |
| CAP13 | Elemicin | $C_{12}H_{16}O_3$ | 487-11-6 | 50 |
| CAP8/10 | Methyl isoeugenol | $C_{11}H_{14}O_2$ | 93-16-3 | 43 |
| CAP5 | Methyleugenol | $C_{11}H_{14}O_2$ | 93-15-2 | 36 |
| CAP23 | Farnesol | $C_{15}H_{26}O$ | 4602-84-0 | 29 |
| CAP20 | beta-Bisabolol | $C_{15}H_{26}O$ | 374-18-2 | 28 |
| CAP4 | Piperitone | $C_{10}H_{16}O$ | 89-81-6 | 27 |
| CAP24 | Bergamotenol | $C_{15}H_{24}O$ | 88034-74-6 | 26 |
| CAP18 | Agarospirol | $C_{15}H_{26}O$ | 1460-73-7 | 24 |
| CAP19 | α-Cadinol | $C_{15}H_{26}O$ | 481-34-5 | 24 |
| CAP16 | (-)-Perillaldehyde | $C_{10}H_{14}O$ | 2111-75-3 | 23 |
| CAP21 | Cedren-13-ol,8- | $C_{15}H_{24}O$ | 18319-35-2 | 21 |
| CAP25 | Lanceol | $C_{15}H_{24}O$ | 10067-28-4 | 21 |

**Table S6:** Core target information table.

| Target | Degree unDir | Betweenness unDir | Closeness unDir |
|---|---|---|---|
| ESR1 | 57 | 1498.326 | 0.004673 |
| SRC | 53 | 1738.005 | 0.004566 |
| PPARG | 49 | 940.6223 | 0.004405 |
| STAT3 | 48 | 766.2441 | 0.004405 |
| EGFR | 47 | 575.4532 | 0.004367 |
| CASP3 | 47 | 509.0006 | 0.004348 |
| PTGS2 | 47 | 1141.988 | 0.004464 |
| MAPK3 | 45 | 648.227 | 0.00431 |

| | | | |
|---|---|---|---|
| EP300 | 41 | 699.6267 | 0.004149 |
| APP | 38 | 477.5457 | 0.004219 |
| DRD2 | 38 | 877.4098 | 0.004082 |
| MAOB | 34 | 670.9166 | 0.003984 |
| CCND1 | 33 | 246.9552 | 0.003984 |
| NR3C1 | 33 | 543.2225 | 0.004115 |
| HTR2A | 32 | 520.7556 | 0.003891 |
| AR | 31 | 360.0163 | 0.003922 |
| MAOA | 30 | 682.7451 | 0.004016 |
| NFE2L2 | 30 | 380.5597 | 0.003922 |
| CYP19A1 | 30 | 623.6088 | 0.003891 |
| CREBBP | 30 | 213.8619 | 0.003906 |
| PPARA | 30 | 242.0775 | 0.003861 |
| JAK2 | 29 | 359.5561 | 0.003922 |
| GRM5 | 27 | 491.9587 | 0.003831 |
| ESR2 | 27 | 211.467 | 0.003846 |
| PARP1 | 26 | 316.6822 | 0.003906 |
| SLC6A4 | 25 | 280.9583 | 0.003788 |
| HMGCR | 25 | 222.4382 | 0.003704 |
| MAPT | 25 | 290.4535 | 0.003817 |
| CRHR1 | 25 | 340.3274 | 0.003817 |
| HDAC2 | 24 | 198.6745 | 0.003571 |
| ACHE | 23 | 280.9758 | 0.003745 |
| HTT | 23 | 180.0514 | 0.003817 |
| CYP1A2 | 22 | 340.9472 | 0.003731 |
| TRPV1 | 22 | 230.5418 | 0.003731 |
| CNR1 | 20 | 186.1177 | 0.003663 |
| GABRA1 | 20 | 226.1431 | 0.003436 |
| CYP2C19 | 18 | 326.1217 | 0.003559 |

**Table S7:** KEGG pathway enrichment results.

| Term | % | Count | PValue | Related genes |
|---|---|---|---|---|
| hsa05207: Chemical carcinogenesis - receptor activation | 28.94737 | 11 | 1.52E-08 | *AR, CCND1, SRC, STAT3, CYP1A2, JAK2, PPARA, ESR1, EGFR, ESR2, MAPK3* |
| hsa05200: Pathways in cancer | 39.47368 | 15 | 1.97E-08 | *CREBBP, HDAC2, STAT3, PTGS2, ESR1, EGFR, ESR2, AR, CCND1, CASP3, EP300, PPARG, JAK2, NFE2L2, MAPK3* |
| hsa04726: Serotonergic synapse | 23.68421 | 9 | 2.52E-08 | *APP, MAOB, MAOA, CASP3, HTR2A, CYP2C19, PTGS2, SLC6A4, MAPK3* |
| hsa04917: Prolactin signaling pathway | 18.42105 | 7 | 4.88E-07 | *CCND1, SRC, STAT3, JAK2, ESR1,* |

| | | | | |
|---|---|---|---|---|
| | | | | *ESR2, MAPK3* |
| hsa05167: Kaposi sarcoma-associated herpesvirus infection | 23.68421 | 9 | 1.44E-06 | *CREBBP, CCND1, SRC, CASP3, STAT3, EP300, JAK2, PTGS2, MAPK3* |
| hsa04919: Thyroid hormone signaling pathway | 18.42105 | 7 | 1.22E-05 | *HDAC2, CREBBP, CCND1, SRC, EP300, ESR1, MAPK3* |
| hsa05203: Viral carcinogenesis | 21.05263 | 8 | 2.37E-05 | *HDAC2, CREBBP, CCND1, SRC, CASP3, STAT3, EP300, MAPK3* |
| hsa04540: Gap junction | 15.78947 | 6 | 3.63E-05 | *GRM5, SRC, HTR2A, DRD2, EGFR, MAPK3* |
| hsa05206:MicroRNAs in cancer | 23.68421 | 9 | 4.51E-05 | *HDAC2, CREBBP, CCND1, CASP3, STAT3, EP300, PTGS2, EGFR, MAPK3* |
| hsa05215: Prostate cancer | 15.78947 | 6 | 5.81E-05 | *AR, CREBBP, CCND1, EP300, EGFR, MAPK3* |
| hsa01522: Endocrine resistance | 15.78947 | 6 | 6.1E-05 | *CCND1, SRC, ESR1, EGFR, ESR2, MAPK3* |
| hsa05161: Hepatitis B | 18.42105 | 7 | 6.37E-05 | *CREBBP, SRC, CASP3, STAT3, EP300, JAK2, MAPK3* |
| hsa04080: Neuroactive ligand-receptor interaction | 23.68421 | 9 | 0.000148 | *GABRA1, GRM5, ADORA2A, CNR1, TRPV1, HTR2A, DRD2, NR3C1, CRHR1* |
| hsa05205: Proteoglycans in cancer | 18.42105 | 7 | 0.000233 | *CCND1, SRC, CASP3, STAT3, ESR1, EGFR, MAPK3* |
| hsa04068: FoxO signaling pathway | 15.78947 | 6 | 0.000241 | *CREBBP, CCND1, STAT3, EP300, EGFR, MAPK3* |
| hsa05417: Lipid and atherosclerosis | 18.42105 | 7 | 0.000301 | *SRC, CASP3, STAT3, PPARG, JAK2, MAPK3, NFE2L2* |
| hsa01521: EGFR tyrosine kinase inhibitor resistance | 13.15789 | 5 | 0.000367 | *SRC, STAT3, JAK2, EGFR, MAPK3* |
| hsa05163: Human cytomegalovirus infection | 18.42105 | 7 | 0.000385 | *CCND1, SRC, CASP3, STAT3, PTGS2, EGFR, MAPK3* |
| hsa04024: cAMP signaling pathway | 18.42105 | 7 | 0.000385 | *CREBBP, ADORA2A, EP300, DRD2, PPARA, CRHR1, MAPK3* |
| hsa05165: Human papillomavirus infection | 21.05263 | 8 | 0.00049 | *HDAC2, CREBBP, CCND1, CASP3, EP300, PTGS2, EGFR, MAPK3* |

**Table S8:** Docking results of target protein and active compound.

| Core target | PDB ID | Binding energy/(kcal $Mol^{-1}$) | |
|---|---|---|---|
| | | beta-Asarone | Elemicin |
| ESR1 | 7UJM | -6.4 | -5.6 |
| SRC | 2SRC | -5.8 | -5.7 |

| | | | |
|---|---|---|---|
| PPARG | 8BF1 | -5.9 | -6.0 |
| STAT3 | 6NUQ | -5.0 | -4.6 |
